# CDCP1 regulates retinal pigmented epithelial barrier integrity for the development of experimental autoimmune uveitis

**DOI:** 10.1172/jci.insight.157038

**Published:** 2022-09-22

**Authors:** Lingjun Zhang, Nozha Borjini, Yu Lun, Sweta Parab, Gospel Asonye, Rupesh Singh, Brent A. Bell, Vera L. Bonilha, Andrei Ivanov, David A. Fox, Rachel Caspi, Feng Lin

**Affiliations:** 1 Department of Inflammation and Immunity, Lerner Research Institute, Cleveland Clinic, Cleveland, Ohio, USA.; 2 Department of Ophthalmic Research, Cole Eye Institute, Cleveland Clinic, Cleveland, Ohio, USA.; 3 Division of Rheumatology and Clinical Autoimmunity Center of Excellence, University of Michigan, Ann Arbor, Michigan, USA.; 4 Laboratory of Immunology, National Eye Institute, NIH, Bethesda, Maryland, USA.

**Keywords:** Autoimmunity, Autoimmune diseases

## Abstract

Cub domain-containing protein 1 (CDCP1) is a protein that is highly expressed on the surface of many cancer cells. However, its distribution in normal tissues and its potential roles in nontumor cells are poorly understood. We found that CDCP1 is present on both human and mouse retinal pigment epithelial (RPE) cells. CDCP1-KO mice developed attenuated retinal inflammation in a passive model of autoimmune uveitis, with disrupted tight junctions and infiltrating T cells detected in RPE flat mounts from WT but not CDCP1-KO mice during EAU development. Mechanistically, we discovered that CDCP1 on RPE cells was upregulated by IFN-γ in vitro and after EAU induction in vivo. CD6 stimulation induced increased RPE barrier permeability of WT but not CDCP1-knockdown (CDCP1-KD) RPE cells, and activated T cells migrated through WT RPE monolayers more efficiently than the CDCP1-KD RPE monolayers. In addition, CD6 stimulation of WT but not the CDCP1-KD RPE cells induced massive stress fiber formation and focal adhesion disruption to reduce cell barrier tight junctions. These data suggest that CDCP1 on RPE cells interacts with CD6 on T cells to induce RPE cytoskeleton remodeling and focal adhesion disruption, which open up the tight junctions to facilitate T cell infiltration for the development of uveitis.

## Graphical abstract

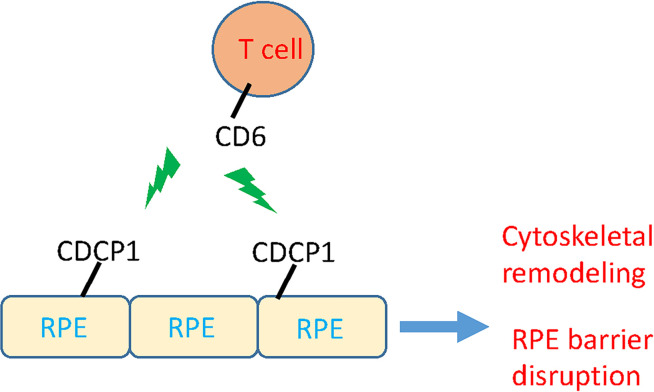

## Introduction

Uveitis, a group of ocular inflammatory diseases that affect approximately 2 million Americans annually, is responsible for an estimated 10%–15% of all cases of severe visual disability (1). Of these diseases, autoimmune posterior uveitis, in which the immune system attacks the retina, is the most sight threatening. Each year, 17.6% of patients with acute uveitis experience transient or permanent blindness, and 12.5% develop other ocular complications such as glaucoma (1). However, the exact causes of autoimmune uveitis remain unknown, and, although the disease can usually be controlled by immunomodulatory treatments, no cure has been identified.

Experimental autoimmune uveitis (EAU) is the best available in vivo model for autoimmune posterior uveitis (2). In EAU, and likely in autoimmune uveitis, research evidence has established that pathogenic T cells autoreactive to retinal antigen(s) are primed in the periphery and migrate through the blood-retina barrier (BRB), which is composed of the retinal pigment epithelium (RPE) and retinal vascular endothelium, to induce retinal inflammation and vision loss (3). The critical role of the retinal vascular endothelium barrier, the inner BRB, in EAU development and its regulatory mechanisms have been extensively studied and relatively established. However, despite existing evidence showing that leukocytes, including autoreactive T cells, also migrate through the outer BRB, also known as the RPE barrier, to induce EAU (4–8), studies on the detailed mechanisms regulating the T cell migration through the outer BRB during EAU development are limited.

CUB domain-containing protein 1 (CDCP1; also known as CD318) is a type I transmembrane protein that is expressed at high levels on many solid tumor cells (9). Most previous studies of CDCP1 have been limited to its role in tumorigenesis and have shown that CDCP1 is critical for cancer cell survival and metastasis through intrinsic mechanisms within the tumor cells (10–12). Few studies have explored the potential roles of CDCP1 on nontumor cells, and therefore, the distribution of CDCP1 under physiological conditions and the potential extrinsic mechanisms through which CDCP1 might act remain unclear.

We recently identified CDCP1 as a novel ligand of CD6, a surface marker and critical regulator of T cells (13). Polymorphisms in *CD6* have been associated with multiple sclerosis (MS) (14–16) and autoimmune uveitis (17), which both share an integral role for autoreactive T cells in pathogenesis. We also previously reported that CD6-KO mice are protected from active immunization-induced EAU and exhibit reduced pathogenic T cell responses and decreased T cell infiltration in the retina (18). In addition, the adoptive transfer of uveitogenic T cells from immunized CD6-KO mice into WT naive mice induces less severe retinal inflammation, compared with the transfer of uveitogenic T cells from immunized WT mice (18). Taken together, these previous findings suggest that CDCP1 could play an important role in the development of EAU, especially if this protein is expressed in the retina.

In this project, we developed mAbs that recognize both mouse and human CDCP1. Using one of these mAbs, we examined the distribution of CDCP1 in the retina via immunofluorescent staining and confirmed the results by Western blotting and flow cytometry analyses of isolated primary cells, immortalized cells, and cell lines. We further investigated uveitis development in WT and CDCP1-KO mice on both C57BL/6 and DBA/1 backgrounds after the adoptive transfer of preactivated uveitogenic WT T cells using various ocular imaging techniques. For mechanistic studies, we investigated CDCP1 expression on RPE cells after inflammatory cytokine stimulations in vitro as well as in naive and EAU mice in vivo. We studied RPE barrier integrity in vivo in both WT and CDCP1-KO mice during EAU development. We also developed CDCP1-knockdown (CDCP1-KD) RPE cells and explored the potential regulatory effect of CDCP1 expression in RPE cells on barrier permeability and T cell migration. These studies are the first to our knowledge to establish the distribution of CDCP1 in the retina and to demonstrate a role of CDCP1 in regulating the development of EAU.

## Results

### Development of new anti-CDCP1 mAbs.

**Figure 1:**
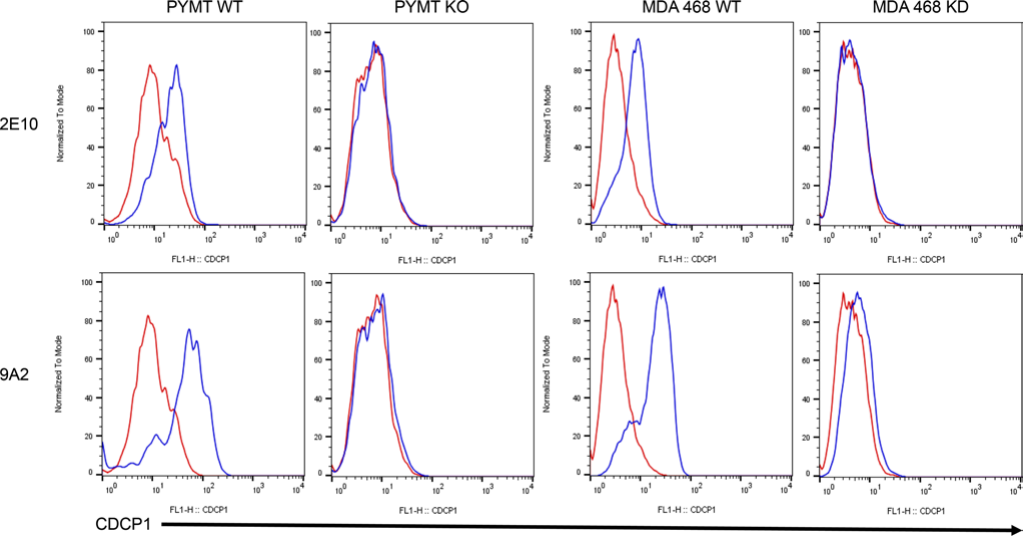
mAb clones 2E10 and 9A2 recognize both mouse and human CDCP1. Clone 2E10 (top row) and clone 9A2 (bottom row) at a concentration of 2 μg/mL selectively labeled mouse PYMT and human MDA 468 WT but not CDCP1-KO/KD cells, respectively. Red lines denote isotype controls; blue lines denote anti-CDCP1 IgGs. Experiments were repeated 3 times. Representative images are presented.

Previous studies have not determined whether CDCP1 is present in the retina and, if so, the distribution in this tissue. Few anti-mouse CDCP1 Abs are available, and none of these cross-reacting Abs yielded satisfactory results in pilot immunofluorescent staining studies (data not shown). To establish the distribution of CDCP1 in the mouse retina, we developed our own anti-mouse CDCP1 mAb by immunizing CDCP1-KO mice with recombinant mouse CDCP1 protein and generating hybridomas following an established protocol (19). The first round of ELISA screening yielded 22 positive clones from approximately 200 hybridomas, which we screened against mouse WT and CDCP1-KO mammary epithelial cells by flow cytometry. Because of the high homology between human and mouse CDCP1, we also subjected the identified anti-mouse CDCP1 mAbs to flow cytometric assays using human WT and CDCP1-KD MDA 468 epithelial cells. Subsequently, we identified 2 mouse anti-mouse CDCP1 mAbs (clones 9A2 and 2E10) that selectively recognized native CDCP1 protein on both human and mouse cell surfaces in our flow cytometry assays (Figure 1).

### CDCP1 is expressed on RPE in the retina.

**Figure 2:**
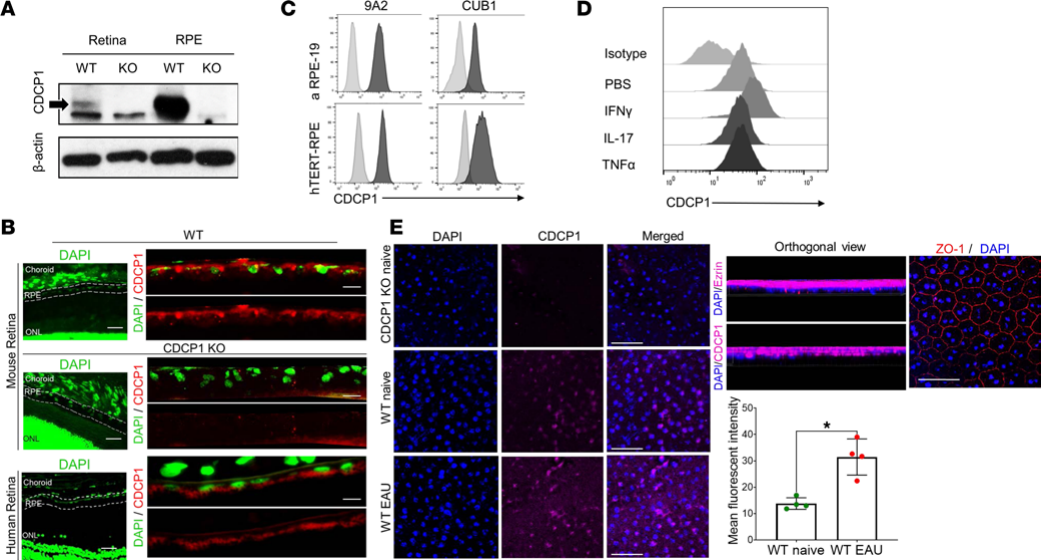
CDCP1 expression and regulation on mouse and human retinal pigment epithelial cells in the retina. (**A**) Retinal tissues and retinal pigment epithelial (RPE) cells were isolated from WT and CDCP1-KO mice, and cell lysates were prepared. CDCP1 was detected by Western blotting with a polyclonal Ab. Here, CDCP1 was detectable in the retina and purified RPE cells from WT but not CDCP1-KO mice. Actin blots were used as loading controls. (**B**) Immunofluorescent staining with the anti-CDCP1 mAb clone 9A2 showed selective CDCP1 expression (red) on RPE cells in mouse and human retinas. DAPI (green) was used for nuclear staining. Scale bars: 10 μm (low-magnification images); 1 μm (high-magnification images). (**C**) CDCP1 expression was detected on human aRPE-19 and hTERT-RPE cells by flow cytometry with both our mAb clone 9A2 and a commercial anti-human CDCP1 mAb, clone CUB1. Gray shades indicate isotype controls; black shades indicate anti-CDCP1 IgGs. (**D**) CDCP1 expression on RPE cells was upregulated by treatment with IFN-γ but not IL-17 or TNF-α. RPE-19 cells were cultured with 1,000 U each of IFN-γ, IL-17, or TNF-α for 48 hours, after which CDCP1 expression was detected using flow cytometry. (**E**) CDCP1 expression on RPE was upregulated in vivo in EAU, as detected by immunofluorescent staining of the RPE flat mounts from WT naive and EAU mice, and using the CDCP1-KO mouse tissue as the negative control. 3D reconstruction and the orthogonal view analyses together with ezrin staining confirmed the surface expression pattern of CDCP1 on the RPE cells. CDCP1 levels (MFI) were quantitated from 4 random areas using Fiji software. Scale bars: 50 μm. Each experiment was repeated at least 3 times.

Given this high homology between human and mouse CDCP1 proteins, we first aimed to determine the presence of CDCP1 in the mouse retina by using a commercially available polyclonal anti-human CDCP1 Ab for Western blotting of prepared and lysed posterior segments of the eyes from WT and CDCP1-KO mice. We detected a CDCP1 band in the WT but not the CDCP1-KO retina tissue lysates, suggesting that CDCP1 is normally present in the retina. We then isolated and lysed primary RPE cells from WT and CDCP1-KO mice and analyzed proteins in the cell lysates by Western blotting. Again, we observed a strong and enriched CDCP1 band in the lysates of RPE cells derived from the WT but not the CDCP1-KO mice (Figure 2A).

To confirm these findings, we used anti-CDCP1 mAb (9A2) to assess the distribution of CDCP1 in the mouse retina via immunofluorescent staining of retinal sections from both WT and CDCP1-KO mice. Consistent with the Western blotting results, CDCP1 was detectable on the RPE layer in the retinas from WT mice but not CDCP1-KO mice (Figure 2B). Interestingly, choroid endothelial cells were CDCP1 negative (Supplemental Figure 1; supplemental material available online with this article; https://doi.org/10.1172/jci.insight.157038DS1). Taken together, these studies established for possibly the first time that CDCP1 is selectively expressed on RPE cells in the mouse retina.

We then extended our studies by staining human retinal cryosections with either the anti-CDCP1 mAb 9A2 or the same concentration of control IgG. As in mice, CDCP1 was detectable on RPE in the human retina. Finally, we analyzed the expression of CDCP1 on aRPE-19 cells, a human RPE cell line, as well as on hTERT-immortalized human RPE cells by flow cytometry with both our anti-CDCP1 mAb (9A2) and a commercially available anti-human CDCP1 mAb (clone CUB1). Both Abs revealed strong expression of CDCP1 on both human RPE lines (Figure 2C).

### CDCP1 expression on RPE is upregulated under inflammatory conditions.

Because the inflammatory cytokines IFN-γ, IL-17, and TNF-α have been implicated in the development of autoimmune uveitis in humans and in mice, we studied the effects of these cytokines on CDCP1 expression on aRPE-19 cells in vitro. Briefly, we cultured aRPE-19 cells in the presence or absence of IFN-γ, IL-17, or TNF-α (all 1,000 U) for 48 hours at 37°C and then analyzed the levels of CDCP1 on the cell surfaces by flow cytometry with our anti-CDCP1 mAb (9A2). We found that the CDCP1 levels were augmented by treatment with IFN-γ but not IL-17 or TNF-α (Figure 2D).

To determine regulation of CDCP1 expression on RPE during EAU in vivo, we prepared RPE flat mounts from WT naive and EAU mice, stained them for zonula occludens-1 (ZO-1) (to demonstrate the RPE monolayer structure) or for CDCP1 with the 9A2 mAb, and then analyzed the samples by confocal microscopy. These assays, including the orthogonal view analyses after 3D reconstruction, once again confirmed that CDCP1 was expressed on the RPE surface in mice and showed that CDCP1 expression on RPE was upregulated under retinal inflammatory conditions in the EAU mice (Figure 2E)

### CDCP1-KO mice on two different genetic backgrounds are protected from EAU after the adoptive transfer of preactivated uveitogenic T cells.

**Figure 3:**
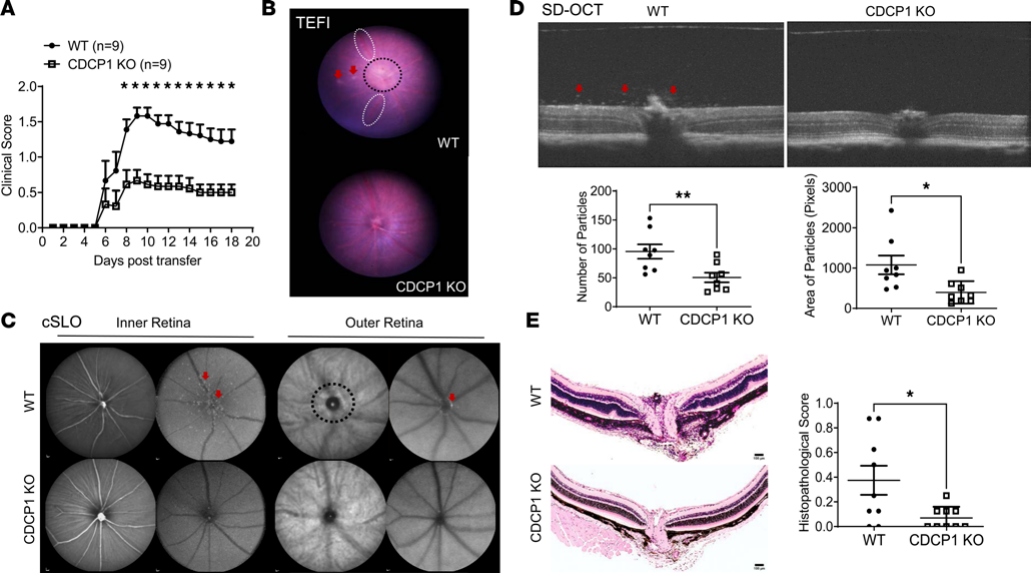
CDCP1-KO mice on a C57BL/6 background develop significantly attenuated EAU after the adoptive transfer of uveitogenic T cells. (**A**) WT and CDCP1-KO mice were adoptively transferred with identical numbers of preactivated uveitogenic WT T cells. CDCP1-KO mice showed decreased clinical scores. *n* = 9 mice for each group. Mean ± SEM; *P < 0.05, 2-way ANOVA. (**B**) Topical endoscopy fundus imaging (TEFI) revealed retinal damages (arrows and black-dot-outlined area) and vasculitis resulting in white sheathing (white-dot-outlined area) caused by EAU in WT mice but not in CDCP1-KO mice. (**C** and **D**) Confocal scanning laser ophthalmoscopy (cSLO) and spectral-domain optical coherence tomography (SD-OCT) revealed more hyperreflective foci (arrows) and lesions (black-dot-outlined area) in WT mice compared with KO mice. Images were taken from all the mice. Representative images are presented. Reflective particles in the vitreous in SD-OCT images were quantified using ImageJ software. Reduced numbers and sizes of particles were observed in KO mice. Each dot represents a mouse. Mean ± SEM; *P < 0.05, **P < 0.01, *t* test. (**E**) Histopathological imaging revealed significantly greater cell infiltration in the vitreous and folds in the retinas of WT mice relative to KO mice and decreased histopathological scores in the latter. Each dot represents a mouse. Mean ± SEM; *P < 0.05, *t* test. Scale bars: 100 μm.

The presence of CDCP1 on RPE cells and its upregulation by IFN-γ in vitro and under EAU conditions in vivo, together with our previous observation that CDCP1 interacted with CD6 on T cells (Figure 2, D and E), suggest that CDCP1 might be important in EAU. To determine the potential role of CDCP1 in the pathogenesis of EAU, we adoptively transferred identical numbers of preactivated uveitogenic WT T cells into both WT and CDCP1-KO mice (C57BL/6 background). We monitored the development of EAU by indirect funduscopy daily and assigned clinical scores accordingly. In some mice, we also evaluated retinal inflammation closely using both spectral-domain optical coherence tomography (SD-OCT) and confocal scanning laser ophthalmoscopy (cSLO). At the end of the experiment (day 18), we collected the eyes from each mouse and assessed the severity of uveitis by histology. Histopathological scores were assigned according to an established protocol. After uveitogenic T cells were adoptively transferred, CDCP1-KO mice developed significantly milder uveitis than WT mice, as indicated by markedly reduced clinical scores based on the indirect funduscopy results (Figure 3A). Similarly, topical endoscopy fundus imaging (TEFI) imaging showed signs of vasculitis and retinal lesions in the WT mice but not the CDCP1-KO mice (Figure 3B), whereas cSLO also revealed abundant inflammatory foci and other retinal abnormalities in the WT mice but not the CDCP1-KO mice after the adoptive transfer of uveitogenic T cells (Figure 3C). Furthermore, SD-OCT examinations found significantly reduced cell infiltration in the retinas of CDCP1-KO mice (Figure 3D). In addition, histological analyses of the retinal sections indicated reduced pathology and significantly lower histopathological scores in CDCP1-KO mice relative to WT mice (Figure 3E). Despite the normal appearance of the RPE layers in the WT EAU mice under the histopathological analyses, immunofluorescent staining of the tight junction protein ZO-1 on the RPE flat mounts prepared from these mice revealed lesions and severe tight junction disruptions (Supplemental Figure 2).

**Figure 4:**
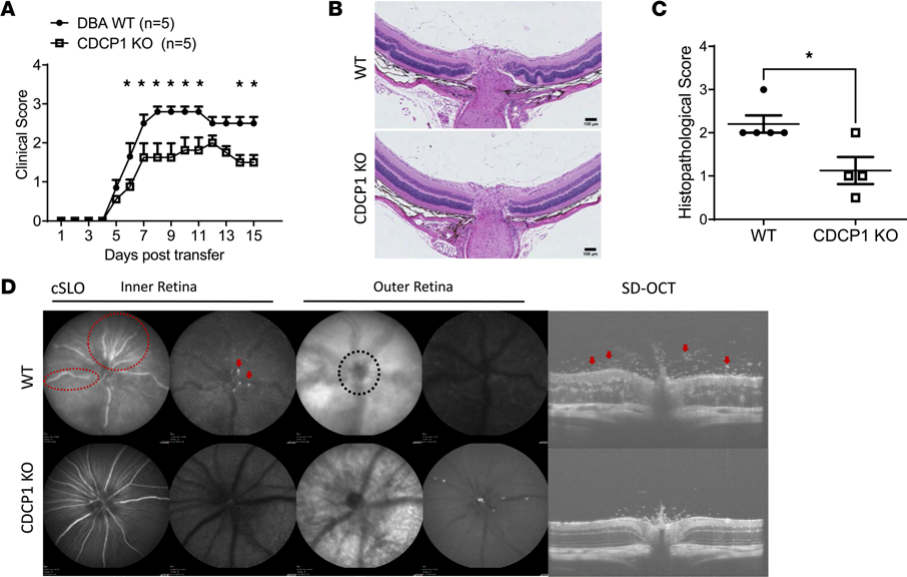
CDCP1-KO mice on DBA/1 background develop significantly attenuated EAU after the adoptive transfer of uveitogenic T cells. (**A**) WT and CDCP1-KO mice were adoptively transferred with identical numbers of preactivated uveitogenic WT T cells. CDCP1-KO mice showed decreased clinical scores. *n* = 5 mice for each group. Mean ± SEM; *P < 0.05, 2-way ANOVA. (**B** and **C**) Histopathological imaging revealed (**B**) greater cell infiltration in the vitreous and folds in the retinas of WT mice relative to KO mice and (**C**) decreased histopathological scores in the latter. Each dot represents a mouse. Mean ± SEM; *P < 0.05, *t* test. Scale bars: 100 μm. (**D**) Confocal scanning laser ophthalmoscopy (cSLO) and spectral-domain optical coherence tomography (SD-OCT) revealed more hyperreflective foci (arrows), lesions (black-dot-outlined area), and swollen retinal vessels (red-dot-outlined area) in WT mice than in KO mice. Images were taken from all the mice. Representative images are presented.

To rigorously confirm discovered role of CDCP1 in EAU, we also adoptively transferred the same numbers of uveitogenic T cells prepared from WT DBA/1 mice into WT and CDCP1-KO mice on the DBA/1 background and assessed the uveitis development using the same ocular imaging techniques. Again, all these assays showed that deficiency of CDCP1 led to attenuated retinal inflammation, with significantly reduced T cell infiltration in the CDCP1-KO mice (Figure 4). Taken together, these studies demonstrated that the CDCP1-KO mice were protected against EAU development in this passive model, thus suggesting a regulatory role of CDCP1 on RPE for T cell migration through the RPE barrier.

### Tight junctions are severely disrupted, with infiltrating T cells detected in RPE flat mounts from WT but not CDCP1-KO mice during EAU development.

**Figure 5:**
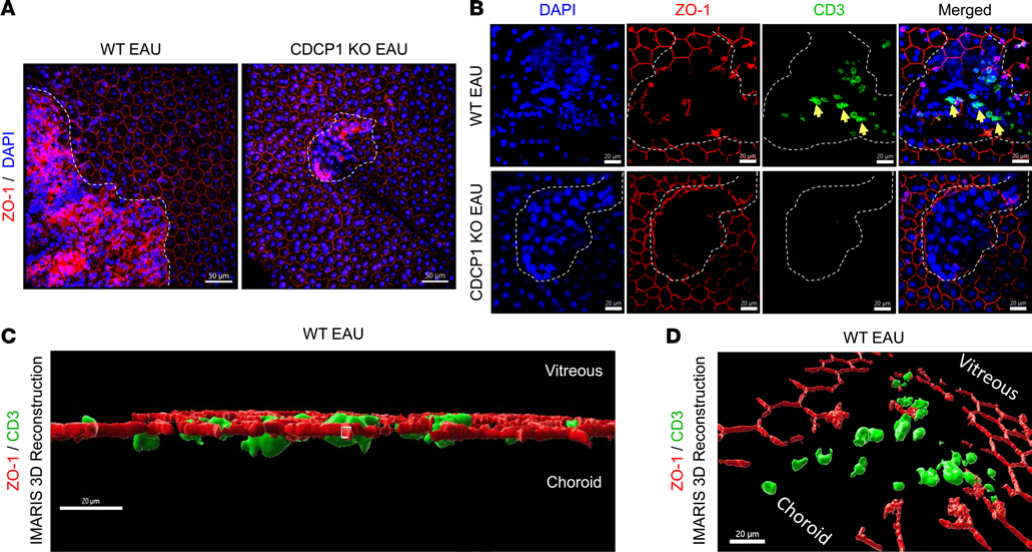
RPE tight junctions are severely disrupted in WT but not CDCP1-KO mice during EAU development. WT and CDCP1-KO mice were adoptively transferred with identical numbers of preactivated uveitogenic WT T cells, and 6 days later, mice were perfused and RPE flat mounts were prepared and stained for ZO-1 and CD3. (**A**) Confocal analyses of the samples showed massive numbers of lesions and RPE tight junction disruptions in WT mice and few lesions and relatively intact tight junctions in the CDCP1-KO mouse RPE flat mounts. Scale bars: 50 μm. (**B**) Further closer analyses also identified infiltrating CD3^+^ T cells (green) only in some of the lesions in the RPE flat mounts from the WT mice. IMARIS 3D reconstruction of ZO-1 and CD3 in WT EAU after background subtraction and surface module rebuild. Scale bars: 20 μm. (**C** and **D**) Representative horizontal orientation showing CD3^+^ T cells migrating through the retina layer. Representative images are presented. Lesions are identified by dotted lines and arrows point to the CD3^+^ T cells. Scale bars: 20 μm.

To evaluate the RPE barrier integrity in WT and CDCP1-KO mice during EAU development, we adoptively transferred WT uveitogenic T cells into these mice and, 6 days later, prepared RPE flat mounts and stained them for ZO-1 and CD3, followed by confocal analyses. These experiments revealed massive numbers of lesions and tight junction disruptions in the RPE flat mounts from the WT mice but not the CDCP1-KO mice after EAU induction (Figure 5A). CD3^+^ T cells were also identified in some of the RPE lesions from WT mice but not CDCP1-KO mice (Figure 5B). More importantly, further analyses of the confocal images and 3D reconstruction demonstrated that at least some of the T cells were migrating through the RPE barrier toward the vitreous (Figure 5, C and D). These results provided direct in vivo evidence suggesting that CDCP1 regulates RPE tight junctions and that T cells transmigrate through the outer BRB during EAU development.

### Development of CDCP1-KD aRPE-19 cells.

**Figure 6:**
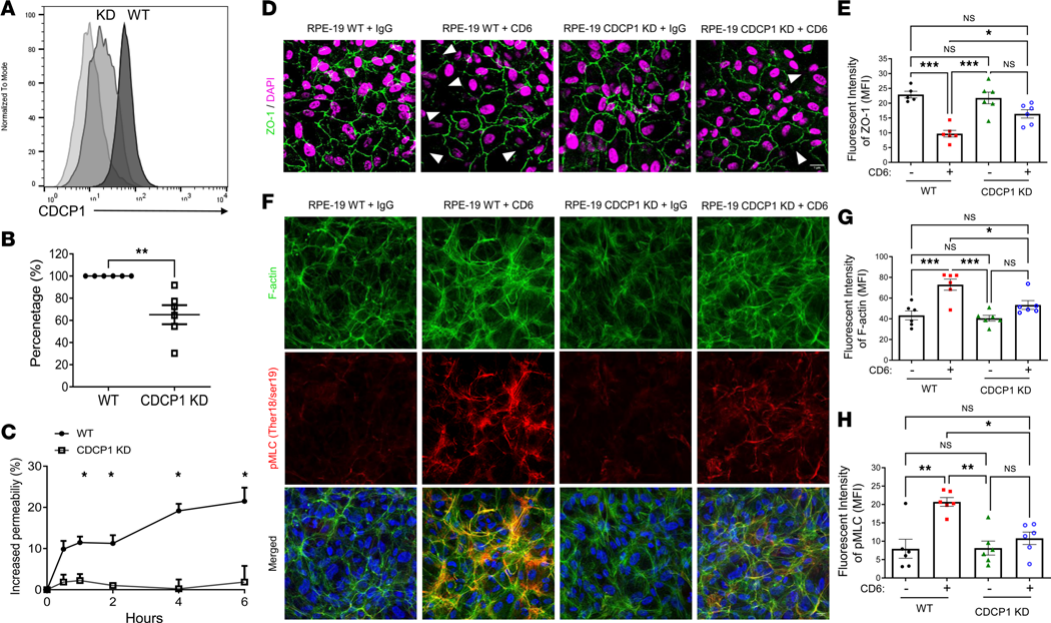
CDCP1 knockdown on RPE cells impairs T cell transmigration by reducing CD6-stimulated cytoskeletal remodeling and tight junction disruption. (**A**) Flow cytometry analysis showed decreased CDCP1 levels on CDCP1-knockdown (CDCP1-KD) RPE cells. (**B**) WT and CDCP1-KD RPE cells were cultured in top chambers of Transwell inserts to form monolayers for T cell migration assays. CFSE-labeled mouse T cells were activated and overlaid on RPE monolayers. After 18 hours, numbers of T cells in the top and bottom chambers were quantified using flow cytometry, and the percentages of migration were calculated. Experiments were repeated 3 times. **P < 0.01, *t* test. (**C**) After CD6 stimulation, permeabilities of WT and CDCP1-KD RPE monolayers were measured using a FITC-dextran leakage assay. CDCP1-KO RPE monolayer had decreased barrier permeability. Experiments were repeated twice. *P < 0.05, 2-way ANOVA. (**D**) WT and CDCP1-KD RPE cells were analyzed by confocal microscopy after a 6-hour incubation with hCD6-Fc or human IgG protein. Representative confocal images of ZO-1 (green) and DAPI (magenta). White arrowheads marked ZO-1 loss. (**E**) CDCP1-KD RPE cells have increased ZO-1 expression compared with WT cells after CD6 treatment. (**F**) Representative images of actin filaments (green) and activated myosin light chain (pMLC, red) in WT and CDCP1-KD RPE cells after a 4-hour incubation with 1 μg/mL recombinant hCD6-Fc fusion protein or human IgG. (**G** and **H**) CD6 treatment induced robust actin filament bundle and stress fiber formation but had a milder effect on the CDCP1-KD cells, as shown by mean fluorescence intensity (MFI). Data were quantitated and analyzed using 2-way ANOVA and Tukey’s test after repeating the experiment 3 times (*n* = 6 images/condition, experiments were repeated twice) and are presented as the mean ± SEM; *P < 0.05, **P < 0.01, ***P < 0.001. Scale bars: 1 μm.

To complement the in vivo studies of CDCP1-KO mice, we used shRNA to develop CDCP1-KD RPE cells for in vitro mechanistic studies. We initially transfected the aRPE-19 cells with lentiviruses coding shRNA specific for the CDCP1 transcript or an empty viral vector (controls) to generate stable transfectants. We then used flow-assisted cell sorting to sort the transfected cells according to their surface expression of CDCP1 to establish WT and CDCP1-KD RPE lines. Ultimately, we generated CDCP1-KD RPE cells on which the surface CDCP1 levels were at least 3-fold lower than those on the WT control aRPE-19 cells (Figure 6A).

### CDCP1-CD6 interaction facilitates T cell transmigration through the RPE monolayer.

The attenuation of EAU development in CDCP1-KO mice after the adoptive transfer of uveitogenic T cells suggests that CDCP1 expression on RPE cells might be required to enable efficient T cell migration through the outer BRB. To test this hypothesis, we first cultured WT and CDCP1-KD aRPE-19 cells on Transwell inserts for 2 weeks to allow monolayer formation. Subsequently, we overlaid activated WT T cells on the RPE monolayers and then measured the migration of the T cells through the RPE monolayers. Through repeated experiments, we confirmed that the ability of activated T cells to migrate through the RPE monolayer was impaired when their CDCP1 levels were reduced, suggesting that CDCP1 on RPE cells facilitates T cell infiltration through the outer BRB (Figure 6B).

### CDCP1-CD6 interaction increases RPE barrier permeability.

One possible mechanism by which CDCP1-KD reduced T cell migration through the RPE barrier could be that CDCP1 on RPE interacts with CD6 on T cells to open the RPE barrier. We thus cultured the WT and CDCP1-KD RPE cells to allow the barrier formation and then stimulated them with the same concentration of CD6. After 6 hours of stimulation, we assessed the barrier integrity using a FITC-dextran leakage-based permeability assay. These studies showed that CD6 stimulation of the WT cells, but not the CDCP1-KD RPE cells, induced significant leakage of the FITC-dextran through the RPE monolayer (Figure 6C), suggesting that CD6 interacts with CDCP1 on RPE cells to open up the barrier.

### CDCP1-CD6 interaction induces RPE cytoskeleton remodeling and tight junction reduction.

To further explore the mechanism by which CDCP1 on RPE cells could increase the outer BRB permeability, we repeated the CD6 stimulation experiments using the WT and CDCP1-KD aRPE-19 cells after their monolayer formations and then analyzed the expression of tight junction protein ZO-1, as well as the stress fiber formation and myosin light chain phosphorylation by confocal microscopy after respective immunofluorescent staining. These experiments showed that, in WT RPE monolayers, CD6 stimulation induced significant stress fiber formation (F-Actin) and myosin light chain phosphorylation in association with decreased ZO-1 expression levels. In contrast, the CDCP1-KO RPE monolayer showed significantly reduced cytoskeleton remodeling and tight junction changes after CD6 stimulation (Figure 6, D–H). These results suggest that CDCP1-CD6 interaction induced RPE cytoskeleton remodeling and tight junction reduction, which increase the RPE barrier permeability.

## Discussion

In this report, we discuss anti-CDCP1 mAb clones that recognize both native human and mouse CDCP1 proteins on the cell surfaces. Using a commercial polyclonal Ab and one of our mAbs, we established the presence of CDCP1 on RPE cells in both mouse and human retinas by Western blotting and/or immunofluorescent staining. We further determined that CDCP1 expression is upregulated by the inflammatory cytokine IFN-γ in vitro and under EAU conditions in vivo. More importantly, CDCP1-KO mice on 2 different genetic backgrounds were protected against the development of passive EAU, in which retinal inflammation is induced by the adoptive transfer of preactivated uveitogenic T cells. Our mechanistic studies using RPE flat mounts from WT and CDCP1-KO mice after EAU induction, as well as WT and CDCP1-KD aRPE-19 cells in various assays, confirmed that CDCP1 on RPE cells is a critical facilitator of T cell migration through the outer BRB by regulating the RPE cytoskeleton remodeling and tight junction after interacting with CD6.

In previous studies, upregulated CDCP1 expression was observed in certain solid tumors and was shown to be associated with a poor prognosis in some patients (20, 21). Even though CDCP1 is known to be expressed on certain epithelial cells (22), fibroblasts (13), mesenchymal cells (23), and some hematopoietic stem cells (24) under physiological conditions, previous studies of this protein have been limited almost exclusively to tumors, and the potential effects of CDCP1 were largely unknown outside of tumor biology. We recently identified CDCP1 as a novel ligand of CD6 (13), a T cell surface marker, and an identified risk gene in MS (15) and autoimmune uveitis (17). In our previous studies of experimental autoimmune encephalomyelitis (EAE), an animal model of MS, we found that CD6-KO mice were protected, as indicated by reduced T cell infiltration and inflammation in the CNS (25). We also observed that CD6-KO T cells migrated through a brain microvascular endothelial cell monolayer less efficiently than WT T cells in vitro, suggesting that CD6 interacts with its ligands (receptors) on the endothelial cells that compose the blood-brain barrier and, thus, facilitates T cell infiltration into the CNS to cause inflammation and disease. CD166, the first identified ligand of CD6, is present on many cells, including endothelial cells and RPE cells (26). However, the functions of CD6 might not completely depend on its interaction with CD166, as CD166 blockade does not influence the in vitro proliferation and activation of human CD4 T cells (27). Furthermore, the therapeutic anti-CD6 mAbs itolizumab and UMCD6 do not interfere with the CD6-CD166 interaction. Nevertheless, although a CD166-blocking mAb could effectively ameliorate EAE by reducing T cell migration into the CNS (27), CD166-KO mice develop exacerbated EAE due to increased permeability of the BBB in the absence of CD166 on microvascular endothelial cells (28).

Autoimmune uveitis occurs in many patients with systemic autoimmunity, such as Behcet’s disease, Vogt Koyanagi Harada’s disease, and systemic sarcoidosis (29). EAU is a model of autoimmune uveitis in which retinal inflammation occurs after subcutaneous active immunization with a peptide from the retinal antigen IRBP. In EAU, pathogenic T cells are primed in the periphery and migrate through the BRB to cause local inflammation and, ultimately, uveitis (29). As noted previously, certain *CD6* polymorphisms are associated with autoimmune posterior uveitis (17), but the underlying mechanism remains unclear. We previously reported that CD6-KO mice exhibit ameliorated retinal inflammation in EAU, with reduced T cell infiltration in the retina both after active immunization and the adoptive transfer of uveitogenic T cells (18). In addition, anti-CD6 mAb treatment significantly reduced retinal T cell infiltration in a mouse model of EAU (18). Taken together, these data indicate strongly that CD6 on T cells might interact with its receptors, such as CDCP1, on the BRB to facilitate retinal infiltration and uveitis development.

Previous studies have not explored the presence and distribution of CDCP1 in the retina and BRB. As noted above, we chose to develop anti-CDCP1 mAbs after determining that the available options were unsatisfactory and selected CDCP1-KO mice for immunization because their immune systems had never “seen” CDCP1; accordingly, this antigen would elicit a strong immune response and avoid potential immune tolerance, which was a concern given the high homology between the mouse and human CDCP1 proteins. We identified 2 mouse anti-mouse CDCP1 mAbs that also cross-react with CDCP1 on the surfaces of human cells in flow cytometric assays. These mouse anti-mouse/human CDCP1 mAbs will not only be useful for analytic studies, such as immunofluorescent staining, as described in this report, but may also yield significant advantages for future in vivo therapeutic studies after their functional neutralization activities are confirmed. Importantly, these mouse IgGs can be administered repeatedly to mouse disease models without immunogenicity that might compromise the studies.

Through our Western blotting analyses of retinal lysates prepared from naive WT and CDCP1-KO mice, we have provided what we believe to be the first evidence of CDCP1 protein expression in the retina. Furthermore, immunofluorescent staining of retinal sections as well as RPE flat mounts from WT and CDCP1-KO mice established the expression of CDCP1 on RPE cells. These findings, together with the other findings showing that CDCP1-KO mice were protected in passive models of EAU suggest that CDCP1 on RPE cells facilitates T cell migration through the BRB to promote retinal inflammation. The observation of an identical distribution of CDCP1 protein expression in the human retina suggests that the mechanisms by which CDCP1 regulates EAU development in mice might also apply to humans with autoimmune uveitis.

The results that RPE flat mounts from WT mice, but not CDCP1-KO mice, showed massive tight junction disruption after EAU induction and that WT, but not CDCP1-KD, RPE monolayers showed increased permeability after CD6 stimulation and allowed improved T cell transmigration suggest that CDCP1 on RPE interacts with CD6 on T cells to regulate the outer BRB barrier function. Our previous studies in EAE using CD6-KO mice and CDCP1-KO mice predict that CDCP1 on RPE might interact with CD6 on T cells like adhesion molecules to facilitate the infiltration of the uveitogenic T cells into the retina for EAU development. However, the results from confocal analyses of F-actin formation, myosin light chain phosphorylation, and ZO-1 expression of WT and CDCP1-KD RPE cells after CD6 stimulation suggest that by interacting with CD6, CDCP1 on RPE cells directly regulates the RPE barrier permeability to facilitate T cell migration through the outer BRB by inducing cytoskeleton remodeling and decreasing tight junction protein expression. These results also suggest that targeting CDCP1 to block CD6-CDCP1 interactions might be a new effective therapeutic approach for EAU and possibly autoimmune uveitis because this approach could help to maintain the integrity of the outer BRB to prevent the infiltration of the uveitogenic T cells into the retina.

As described in the Introduction, activated uveitogenic T cells migrate through both the inner and outer BRBs to elicit EAU. Our confocal analyses and 3D reconstruction results also clearly demonstrated that T cells do migrate through the RPE barrier during EAU development. In fact, activated T cells of any specificity probably routinely cross the BRB but do not elicit uveitis unless they recognize their cognate antigen (30, 31). Even though we did not detect CDCP1 expression on choroidal vascular endothelial cells in the eye sections examined, and all the data support the notion that CDCP1 on RPE interacts with CD6 on T cells to open up the outer BRB to facilitate T cell transmigration, the possibility of CDCP1 regulating the inner BRB during EAU development is plausible, especially in the light of our previous report showing that CDCP1 expression is inducible on mouse brain microvascular endothelial cells in EAE (13); the retinal cross-sections that we studied are not the best suitable to examine retinal vasculatures. Furthermore, our data do not address the question of at what point relative to the inner BRB does the outer BRB become breached to facilitate T cell migration and thus uveitis development. That said, the observation that expression of CDCP1 on RPE is enhanced under inflammatory conditions could suggest that breakdown of the inner BRB and onset of inflammation precede breakdown of the outer BRB. Future time-course studies using retinal flat mounts, RPE flat mounts, and retinal endothelium– and RPE-specific CDCP1-KO mice are warranted to further address these important possible regulatory pathways.

In summary, we have developed what we believe to be novel anti-CDCP1 mAbs that recognize both human and mouse CDCP1 and established the distribution of CDCP1 in both mouse and human retinas. We further discovered that CDCP1 is required for the development of passive EAU in mice on 2 different genetic backgrounds, potentially by regulating RPE barrier function through modulation of cytoskeleton remodeling to facilitate T cell migration through the outer BRB. These data not only demonstrate the potentially novel immunoregulatory function of CDCP1 beyond its established role in tumor biology, but also suggest that CDCP1 may be a new therapeutic target in human autoimmune uveitis.

## Methods

### Animals.

C57BL/6J WT and CDCP1-KO mice were obtained from The Jackson Laboratory. Sex- and age-matched (8–12 weeks of age) mice were used for all experiments. These CDCP1-KO mice (C57BL/6 background) were also back-crossed with DBA/1 mice (The Jackson Laboratory) for 13 generations to generate the congenic CDCP1-KO mice on the DBA/1 background. Mice were maintained under pathogen-free conditions in the animal facilities of the Cleveland Clinic Lerner Research Institute.

### Induction of EAU by adoptive transfer of uveitogenic T cells.

Passive EAU was induced by preactivated uveitogenic T cells according to a previously published protocol. First, WT C57BL/6J and DBA/1 mice were immunized by subcutaneous injection of 200 μg human IRBP_651–670_ peptide (C57BL/6J) and IRBP_161–180_ peptide (DBA/1) (GenScript USA Inc.), respectively, and 250 μg *Mycobacterium tuberculosis* H37Ra in complete Freund’s adjuvant (Difco Laboratories Inc.). After 14 days, splenocytes and draining lymph node cells from immunized mice were cultured in the presence of 10 μg/mL of the respective IRBP peptide and 10 ng/mL IL-23 in complete RPMI 1640 media (supplemented with 10% FBS) for 72 hours. Uveitogenic T cells were separated by density centrifugation over a Ficoll gradient and injected intraperitoneally into each WT and CDCP1-KO mouse (5 × 10^6^ T cells/C57BL/6J mouse and 3 × 10^6^ T cells/DBA/1 mouse)

### Development of mouse anti-mouse/human CDCP1 mAbs.

CDCP1-KO mice were immunized with a recombinant mouse CDCP1 protein (R&D Systems), and hybridomas were generated from the immunized mice following an established protocol (32). The culture supernatants were screened by ELISA, and the positive clones were screened against WT and CDCP1-KO mouse epithelial cells (33) or WT and CDCP1-KD human epithelial cells (34) in flow cytometric analyses.

### Immunofluorescent staining of CDCP1 in the WT and CDCP1-KO mouse retinas.

Mice were perfused with 10 mL ice-cold 0.01 M PBS, followed by 20 mL ice-cold 4% paraformaldehyde (PFA). The eyes were removed and post-fixed with 4% PFA for 24 hours and then washed with 30% sucrose solution until the tissue sank. Subsequently, the eyes were embedded in SD-OCT medium (Thermo Fisher Scientific) and kept at −80°C until processed. Coronal sections (14 μm thickness) were prepared and processed for morphological studies. For immunohistochemistry, the sections were first rehydrated and then incubated for 1 hour in PBS containing 0.3% Triton X-100, 10% normal donkey serum, and 1% BSA, followed by incubation with a mouse anti-CDCP1 primary Ab (clone 9A2) diluted in the preabsorption solution (dilution, 1:500) overnight at 4°C. After 3 washes in PBS, the sections were incubated with a secondary Ab anti-mouse IgG conjugated with FITC (Jackson ImmunoResearch) for 2 hours. The sections were then mounted with Fluoromount-G (Abcam). Images were taken using a BZ-X700 microscope (Keyence) and SP8 confocal microscope (Leica Microsystems).

For CDCP1 detection in EAU mice, sex- and age-matched naive WT and EAU mice (14 days after passive EAU induction) and matched CDCP1-KO mice were perfused with 10 mL ice-cold PBS, followed by 10 mL ice-cold 4% PFA. The eyes were removed and fixed with 4% PFA for 2 hours at room temperature and then transferred to PBS. RPE flat mounts were dissected according to a published protocol (35), stained with 9A2, and analyzed by confocal microscopy, following the protocol described above with minor modifications. After acquisition the orthogonal (*xz* plane) view of the images was generated using Imaris software. Mean fluorescence intensity (MFI) of CDCP1 was quantitated using Fiji software (https://imagej.net/). The corrected total fluorescence that we defined as MFI was calculated by subtracting out the background signal by using the following formula: MFI = integrated density – (area × mean fluorescence of background readings)

### Immunofluorescent staining of CDCP1 in the human retina.

Human donor eyes were obtained through the Foundation for Fighting Blindness Eye Donor Program (Columbia, Maryland, USA). Donor eyes were not clinically diagnosed with a retinal degeneration. Human retinal tissues were fixed with 4% PFA and 0.5% glutaraldehyde in Dulbecco’s PBS (D-PBS). Pieces of retina-RPE-choroid were then fixed in 4%PFA, infiltrated with 10%–20% sucrose in PBS, incubated in SD-OCT medium, placed in base molds filled with SD-OCT, frozen rapidly in liquid nitrogen, and kept at −80°C until processed. Cryosections (10 μm) were collected on an HM 505E cryostat (Microm) equipped with a CryoJane Tape-Transfer system (Leica). In brief, slides were pretreated with citrate buffer (pH 6.0; MilliporeSigma) in a microwave for 2 minutes, followed by blocking with 0.1% Triton X-100 and 10% donkey serum in PBS for 2 hours. The slides were then incubated with the primary Ab (mouse anti-CDCP1, 9A2; dilution, 1:500) in PBS with 0.1% Triton X-100 and 2% donkey serum overnight at room temperature, washed 3 times in PBS, and incubated with the secondary Ab (FITC-conjugated anti-mouse IgG, Jackson ImmunoResearch; dilution, 1:500) for 2 hours. The sections were mounted with Fluoromount-G (Abcam) and treated with TrueBlack Lipofuscin Autofluorescence Quencher (Biotium) to suppress tissue autofluorescence. Images were taken using a BZ-X700 microscope and SP8 confocal microscope.

### RPE tight junction analysis in the WT and CDCP1-KO mouse retina during EAU development.

EAU was induced in WT and CDCP1-KO mice by adoptive transfer of uveitogenic T cells as described above. On day 6, these mice were perfused with 10 mL of ice-cold PBS followed by 10 mL of ice-cold 4% PFA. The eyes were removed and fixed with 4% PFA for 2 hour at room temperature and then transferred to PBS. RPE flat mounts were dissected according to a published protocol. The RPE flat mounts were treated with 100% acetone for 10 minutes at –20°C, washed twice with PBS and blocked with PBS containing 0.3% Triton X-100, 2% normal goat serum and 3% BSA for 2 hours at room temperature. This was followed by incubation with a ZO-1 Ab (61-7300; Invitrogen; dilution, 1:100), Alexa Flour 647–CD3 Ab (557869; BD Biosciences; dilution, 1:100) overnight at 4°C. After 3 washes in PBS, the RPE flat mounts were incubated with Alexa Flour 594 goat anti-rabbit secondary Ab (111-585-144, Jackson ImmunoResearch; dilution, 1:500) for 2 hours at room temperature. The sections were then mounted with Fluoromount-G (Abcam). Images were taken using a SP8 confocal microscope. 3D reconstruction was done using the IMARIS software package (Oxford Instruments) following the manufacturer provided protocols.

### Development of CDCP1-KD RPE cells.

CDCP1 expression in aRPE-19 cells (ATCC) was knocked down using short hairpin RNA–encoding (shRNA-encoding) lentiviruses (MilliporeSigma). Briefly, 5 × 10^4^ aRPE-19 cells were seeded in a well of a 12-well plate and incubated overnight. The supernatant was removed, and the cells were incubated with fresh media containing 8 μg/mL hexadimethrine bromide and 1 × 10^5^ lentiviral particles encoding CDCP1-specific or nontarget shRNA (MOI = 2) for 18 hours. Successfully transduced cells were further selected by culturing with 2 μg/mL puromycin for 2 weeks, followed by flow cytometric sorting based on CDCP1 expression. Cells were maintained in complete DMEM media supplemented with 10% FBS.

### T cell migration assay.

Initially, 5 × 10^4^ CDCP1-KD and control aRPE-19 cells were cultured in 24-well inserts for 2 weeks until monolayers had formed. The culture medium was replaced every 3 days. WT mouse T cells were isolated from spleens using nylon wool and labeled with CFSE. Subsequently, 1 × 10^5^ purified mouse T cells were seeded into each upper chamber and stimulated with 2 μg/mL anti-mouse CD3 and anti-mouse CD28. Mouse CCL-2 (50 ng/mL) was added to the lower chambers as a chemoattractant. After 18 hours, T cells in the upper and lower chambers were counted using a flow cytometer. The percentage of migration was calculated as (cell count_lower_ _chamber_/cell count_upper_ _chamber_) × 100% and normalized.

### Outer BRB permeability assay.

5 × 10^4^ CDCP1-KD and control aRPE-19 cells were cultured in 24-well inserts for 2 weeks to form monolayers. 1 μg/mL hCD6-Fc (R&D Systems) or IgG (Sino Biological) was incubated with RPE cells for 6 hours. Then, culture medium was changed into the phenol-red free complete DMEM medium with 1 mg/mL of 10 KD FITC-Dextran (MilliporeSigma) in the upper chamber of the insert. 10 μL of the medium in the lower chambers was collected at different time points and diluted at 1:10 with PBS. Fluorescence intensity was measured on a plate reader using 488/520 nm excitation/emission.

### Immunofluorescent staining of RPE cells.

For tight junction ZO-1 labeling, control aRPE and CDCP1-KD cells in culture medium were allowed to adhere to coverslips till confluency in a humidified incubator at 37°C and 5% CO_2_. Two weeks after confluency cells were treated with 1 μg/mL hCD6-Fc fusion protein or human IgG at 37°C for 6 hours and fixed with 100% methanol for 15 minutes at –20°C. After washes, coverslips were blocked for 20 minutes with 1× PBS containing 0.1% Triton X-100, 5% normal donkey serum, and 1% BSA, followed by overnight incubation with the primary Ab rabbit anti-ZO-1 (Thermo Scientific; dilution, 1:50) at 4°C and Alexa Fluor 594 donkey anti-rabbit (Jackson ImmunoResearch; dilution, 1:800) as a secondary Ab for 2 hours at room temperature. Fluoromount-G containing DAPI was used to mount the coverslips on slides.

For cytoskeletal labeling, coverslips were fixed with 4% PFA for 15 minutes at room temperature, permeabilized with 0.1% Triton X-100 in PBS for 15 minutes, blocked with 1× PBS containing 5% normal donkey serum and 1% BSA for 30 minutes, and incubated overnight at 4°C with anti-pMLC (ser18/Thr19) Ab (1:500). After washing, the cells were incubated for 1 h at room temperature with an Alexa Fluor 594 donkey anti-rabbit (1:800) secondary Ab and Alexa Fluor 647 phalloidin (Thermo Scientific; dilution, 1:1,000) to label F-actin. Finally, the coverslips were mounted with Fluoromount-G containing DAPI. For all experiments, images were taken using Leica SP8 confocal microscope. MFI of ZO-1, F-actin, and pMLC was measured using Fiji software. The corrected total fluorescence that we defined as MFI was calculated by subtracting out the background signal by using the following formula: MFI = integrated density — (area × mean fluorescence of background readings).

### Clinical and histopathological scoring.

The mice were examined daily for clinical signs of EAU using a binocular indirect ophthalmoscope (Keeler Instruments Inc.). Disease severity was scored on a scale of 0–4 in a masked fashion, according to published criteria (36). For histopathological scoring, whole eyes were collected on day 18, fixed in 10% neutral buffered formalin, and embedded in paraffin. Five-micrometer sections were cut through the pupil and optic nerve axis and stained with H&E. The sections were assigned histopathological scores of 0–4 based on previously published criteria (36).

### Ocular imaging.

TEFI, cSLO, and SD-OCT were performed as previously described after general anesthesia (37). Briefly, TEFI was performed using a custom-fabricated apparatus containing a 3-mm endoscope and D90s digital SLR camera (Nikon). cSLO imaging (Heidelberg Retina Angiograph II, Heidelberg Engineering) was collected in the infrared-cSLO (λ = 815 nm illumination/reflection) and visible autofluorescence-cSLO (λ = 488 nm excitation/500–680 nm emission) modes. SD-OCT (840 HR SDOIS, Bioptigen Inc.) imaging was used to observe the posterior segment of the eye. All SD-OCT scans had the optic nerve in the center. For each eye, images of horizontal and vertical line scans were analyzed using ImageJ (NIH) to quantify the hyperreflective foci in the vitreous chamber area.

### Statistics.

Statistical analyses were performed using GraphPad Prism 9. Data are expressed as mean ± SEM. Two-tailed Student’s *t* test was used to analyze data from 2 groups. Two-way ANOVA followed by Bonferroni’s test or Tukey’s test, as appropriate, was used to analyze data with 2 independent variables. *P* values of less than 0.05 were considered significant.

### Study approval.

All animal care and experimental procedures were approved by the Institutional Animal Care and Use Committee of Cleveland Clinic and conformed to the US Department of Health and Human Services *Guide for the Care and Use of Laboratory Animals* (National Academies Press, 2011). Immunohistochemical and histological analysis of human eyes were performed with the approval of the Cleveland Clinic Institutional Review Board (IRB 14-057). The research adhered to the tenets of the Declaration of Helsinki.

## Author contributions

LZ, NB, YL, PS, GA, RS, and BAB did experiments, analyzed data, and edited the manuscript. VLB provided human retina samples and analyzed data. AI, DAF, and RC discussed the results and edited the manuscript. FL designed the experiments and prepared the manuscript.

## Supplementary Material

Supplemental data

## Version 1 08/11/2022

In-Press Preview

## Version 2 09/22/2022

Electronic publication
