# Supplementary figures and images for: CDCP1 regulates retinal pigmented epithelial barrier integrity for the development of experimental autoimmune uveitis
Source: JCI Insight. 2022 Sep 22;7(18):e157038. doi: 10.1172/jci.insight.157038 (PMC9675461; doi:10.1172/jci.insight.157038)

## Slide 1
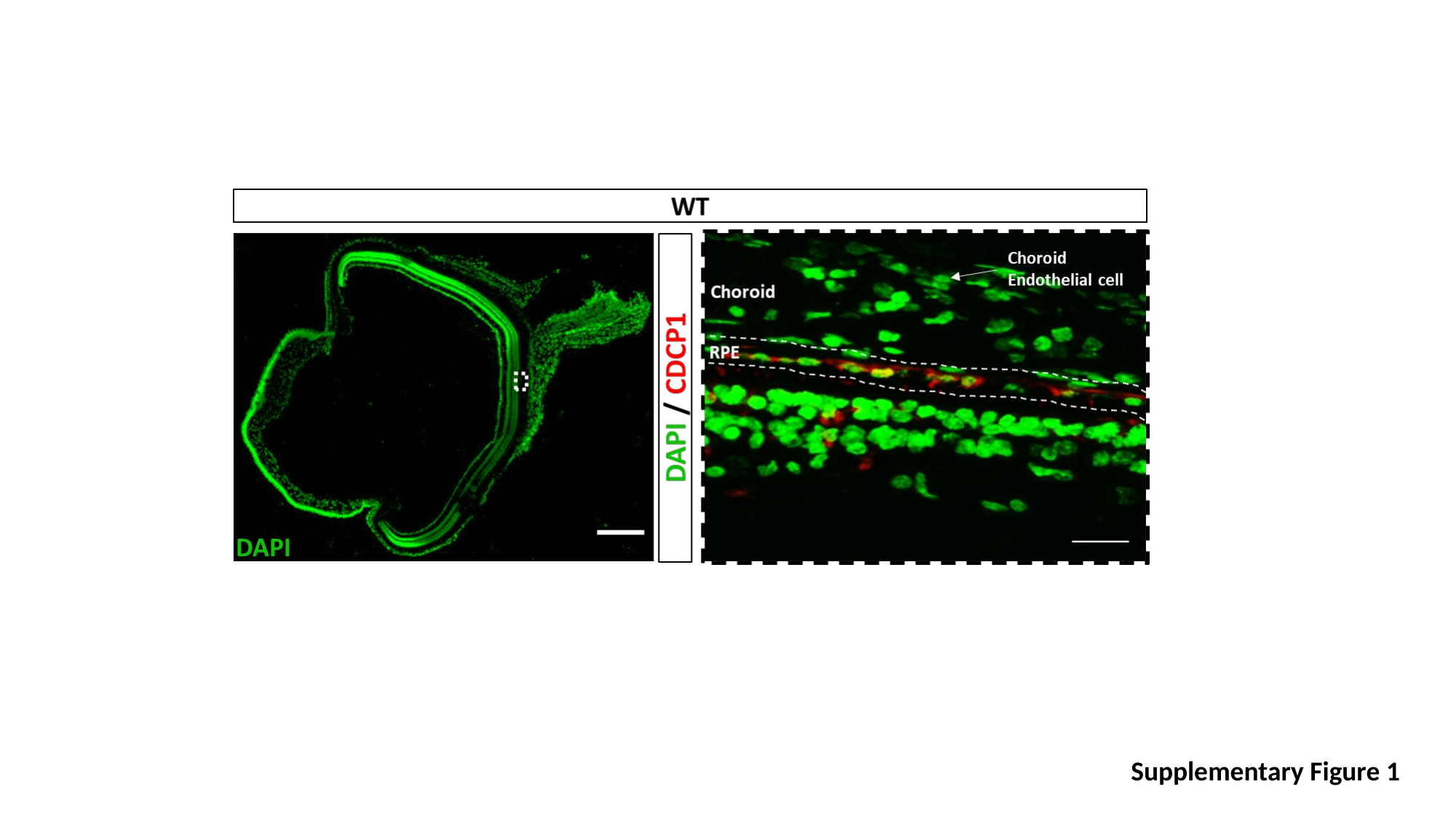

Supplementary Figure 1

## Slide 2
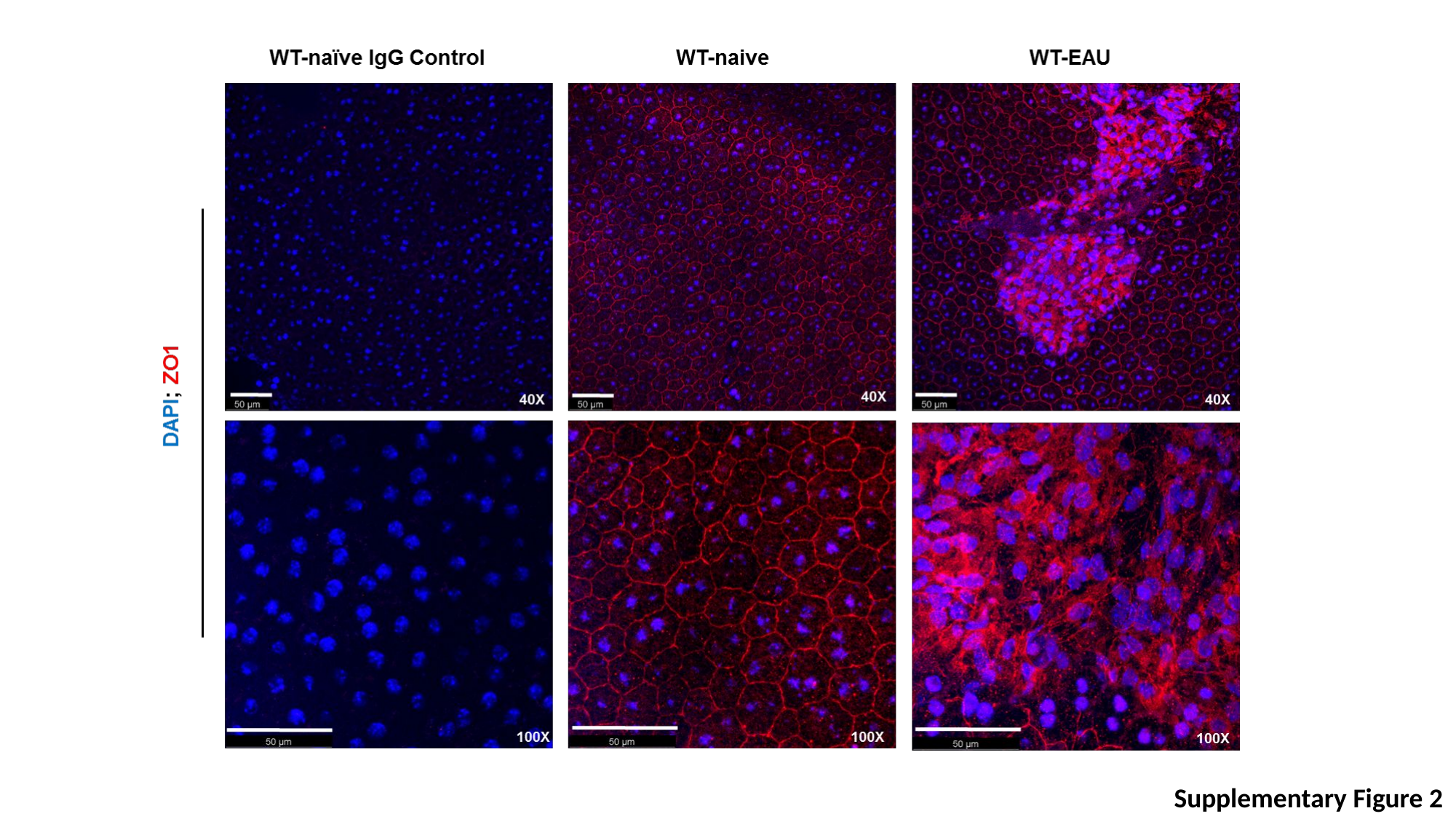

Supplementary Figure 2

Supplement: Supplemental data [file jciinsight-7-157038-s134.pptx]
